# Brain derived neurotrophic factor expression and DNA methylation in response to subchronic valproic acid and/or aldosterone treatment

**DOI:** 10.3325/cmj.2019.60.71

**Published:** 2019-04

**Authors:** Katarina Buzgoova, Jan Graban, Lucia Balagova, Natasa Hlavacova, Daniela Jezova

**Affiliations:** 1 Laboratory of Pharmacological Neuroendocrinology, Institute of Experimental Endocrinology, Biomedical Research Center, Slovak Academy of Sciences, Bratislava, Slovakia; 2 Department of Pharmacology and Toxicology, Faculty of Pharmacy, Comenius University, Bratislava, Slovakia

## Abstract

**Aim:**

To test the hypothesis that valproic acid treatment positively affects brain-derived neurotrophic factor (BDNF) expression and DNA methylation in the hippocampus and brain cortex of rats simultaneously treated with aldosterone.

**Methods:**

Male Sprague-Dawley rats (N = 40) were treated for two weeks with valproic acid (100 mg/1 kg body weight/day) in drinking water and aldosterone (2 μg/100 g body weight/day) or placebo via subcutaneous osmotic minipumps.

**Results:**

Treatment with valproic acid did not modify BDNF gene expression in the hippocampus but reduced BDNF mRNA levels in the brain cortex. Valproic acid treatment marginally enhanced global DNA methylation in the frontal cortex. BDNF expression negatively correlated with DNA methylation in the hippocampus of valproic acid-treated rats. An unexpected finding was that aldosterone treatment significantly decreased global DNA methylation in the hippocampus.

**Conclusion:**

The effect of valproic acid on BDNF expression in the brain may depend on the extent of pathological changes present at the time of treatment onset. The observed negative correlation between BDNF expression and DNA methylation in the hippocampus of valproic acid-treated rats encourages further studies.

Valproic acid has been clinically used for a long time, mainly as an antiepileptic drug and a mood stabilizer. However, we still have not discovered the whole spectrum of its effects and we do not know its exact mechanism of action. There is a clear overlap between epilepsy and psychiatric disorders, and several antiepileptic drugs exert positive effects on mood and anxiety (1). This also applies to valproic acid. The antidepressant effects of valproic acid have been attributed to its ability to inhibit histone-deacetylase and thus induce important epigenetic modulations (2). Neuroprotective action of valproic acid was reported also in animal models of Parkinson’s disease (3), other neurodegenerative diseases (4), and brain ischemia (5).

Neurobiological mechanisms involved in brain plasticity, neuroprotection, and memory processes involve brain growth factors and epigenetic modifications. Growth factors in general, and brain derived neurotrophic factor (BDNF) in particular, play critical roles in the brain by regulating neuronal differentiation and survival, as well as components of synaptic plasticity, such as synaptogenesis or long-term potentiation (6). Cultured rat cortical neurons showed a higher gene expression of BDNF when treated with valproic acid (7). Most of the animal studies showed positive effects of valproic acid on brain plasticity and cognition (8), although some suggested negative effects. Interestingly, valproate also had negative effects on cognition, which was attributed to its histone-deacetylase inhibitory activity. However, the full mechanisms involved are far from being understood (9,10). Memory deficits observed in rats treated with valproic acid were accompanied by decreased cell proliferation and reduced BDNF expression in the hippocampus (9,11).

Though valproic acid is a well-known modulator of epigenetic mechanisms via inhibition of histone-deacetylase (10), only scarce data point to its action on DNA methylation in neuroblastoma cell lines, rodents, and patients with epilepsy (12-15). In general, the role of DNA methylation as a target of psychotropic drug actions is little understood (16).

So far, no study has simultaneously assessed the effects of valproic acid on BDNF expression, DNA methylation, and their potential relationship in an animal model of depression. The aim of the present study is to test the hypothesis that the subchronic treatment with valproic acid positively affects BDNF expression and DNA methylation in the hippocampus and brain cortex of rats simultaneously treated with aldosterone, which was shown to be depressogenic (17).

## MATERIAL AND METHODS

### Animals

The experiments involved 40 male Sprague-Dawley rats (Velaz, Prague, Czech Republic) weighing 250-275 g at the beginning of the experiment. The rats were allowed to habituate to the housing facility for 2 weeks. They were kept under standard housing conditions with a constant 12:12 h light/dark cycle (lights on at 06.00 h), temperature (22 ± 2°C), and humidity (55 ± 10%). Animals were housed individually in standard cages with free access to rat chow and water. All experimental procedures were approved by the Animal Health and Animal Welfare Division of the State Veterinary and Food Administration of the Slovak Republic (Permission No. Ro 2582/14-221). The studies were conducted in 2017.

### Study design

Animals were assigned into the following groups (n = 10 rats/group): 1) vehicle-placebo group; 2) vehicle-valproic acid group; 3) aldosterone-placebo group; and 4) aldosterone-valproic acid group. Aldosterone (*d*-aldosterone, A9477, Sigma Aldrich, St. Louis, MO, USA) or vehicle were continuously administered via osmotic minipumps for 14 days (Model 2002, Alzet, Alza Corp., Cupertino, CA, USA). The number of animals was determined based on our previous studies, in which the number of 10 animals per group was adequate to show the effects of aldosterone treatment on hippocampal gene expression (17). Osmotic minipumps were subcutaneously implanted under isoflurane anesthesia (Forane, Abbott, v.z, Prague, Czech Republic). The aldosterone concentration used to fill the pumps was calculated based on the mean pump infusion rate provided by the manufacturer (0.5 μL/h), animals’ body weight, and the intended dose. The minipumps delivered aldosterone at the dose of 2 μg/100 g body weight/day. The aldosterone dose was chosen based on our previous studies demonstrating anxiogenic and depressogenic effects (17,18). Control animals received minipumps that contained vehicle (1% ethanol solution) only. Aldosterone solubilization and implantation of osmotic minipumps was described previously (18). Valproic acid sodium salt (Sigma Aldrich) was dissolved in distilled water and was administered in drinking water at the dose of 100 mg/1 kg body weight/day continuously for 14 days. Drinking water was renewed every 2 days. Animals from the placebo groups received tap water without valproic acid.

### Tissue collection

Rats were decapitated immediately after they performed the forced swim test. The brain was quickly removed from the skull. The frontal cortexes and hippocampi were quickly removed, frozen in liquid nitrogen, and stored at -80°C until analysis.

### Global DNA methylation analysis

DNA was extracted from the right frontal cortex and the right hippocampus by TRIzol® Reagent (Life Technologies, Carlsbad, CA, USA) following the manufacturer´s instructions. Global DNA methylation was measured using the MethylFlash^TM^ Methylated DNA Quantification Kit (Fluorometric, Epigentek Group Inc., Farmingdale, NY, USA). In this assay, DNA is bound to strip wells that are specifically treated to have a high DNA affinity. The methylated fraction of DNA is detected using capture and detection antibodies and quantified colorimetrically by reading the absorbance in a microplate spectrophotometer. The amount of methylated DNA is proportional to the optic density intensity measured. The total methylation level was assessed by generating a standard curve from Epigentek´s methylated DNA standard.

### BDNF gene expression analysis

BDNF gene expression was measured by real-time polymerase chain reaction (PCR) in the samples of the left frontal cortex and left hippocampus. The total mRNA was isolated by TRIzol® Reagent according to manufacturer’s protocol. The concentration and purity of mRNA preparations was measured by absorption spectroscopy (Nanodrop 2000, Thermo Fisher Scientific Inc., Waltham, MA, USA). One microgram of total RNA was transcribed using oligo (dT) nucleotids by M-MuLV reverse transcription system (ProtoScript, First Strand cDNA Synthesis Kit NewEngland Biolabs, USA). BDNF mRNA concentrations were analyzed by real-time qPCR performed on a Fast Real-Time PCR System 7900 HT (Applied Biosystems, Foster City, CA, USA) using GoTaq Master Mix (Promega, Madison, WI, USA). A specific primer (exon 9) was designed by Primer BLAST NCBI program (*https://www.ncbi.nlm.nih.gov/tools/primer-blast/*) (Table 1). Quantitative PCR reaction was performed using reaction buffer GoTaq qPCR Sybr Green Master Mix (Promega). Primers (Table 1) were used at the concentration of 0.25 pmol/μL. Further analysis steps were performed as described previously (19,20). All data obtained by qPCR analysis were evaluated as nanograms of mRNA (cDNA) according to a standard curve and were normalized to gene expression of *peptidyl prolyl isomerase A* (*PPIA*) and *TATA-Box Binding Protein* gene (*TBP*) as reference genes. This was calculated as a ratio between the quantity of measured gene and the geometric mean of the quantities of reference genes (21).

**Table 1:** Nucleotide sequence of primers used for quantitative polymerase chain reaction

Gene	Forward primer 5′- 3′	Reverse primer 5′- 3′
*Brain-derived Neurotrophic Factor*	ACCATAAGGACGCGGACTTG	AGCAGAGGCTCCAAAGG
*Peptidyl prolyl Isomerase A*	AAGCATACAGGTCCTGGCATCT	CATTCAGTCTTGGCAGTGCAG
*TATA-Box Binding Protein*	TTCGTGCCAGAAATGCTGAA	GTTCGTGGCTCTCTTATTCTCATG

### Statistical analysis

All data sets were normally distributed (as revealed by the Shapiro-Wilk's test) and had homogenous variances (as revealed by the Levene’s test). Therefore, the assumptions for parametric factorial ANOVA were met. Data were analyzed by two-way analysis of variance (ANOVA) with valproic acid (valproic acid and placebo groups) and aldosterone (aldosterone and vehicle groups) as factors. Results are expressed as mean ± standard deviations. Values were winsorized when necessary. The Pearson correlation was used to assess the relationships between the parameters. The level of significance was set at *P* < 0.05. The analyses were performed using Statistica 10 software (StatSoft Inc., Tulsa, OK, USA).

## RESULTS

**Figure 1:**
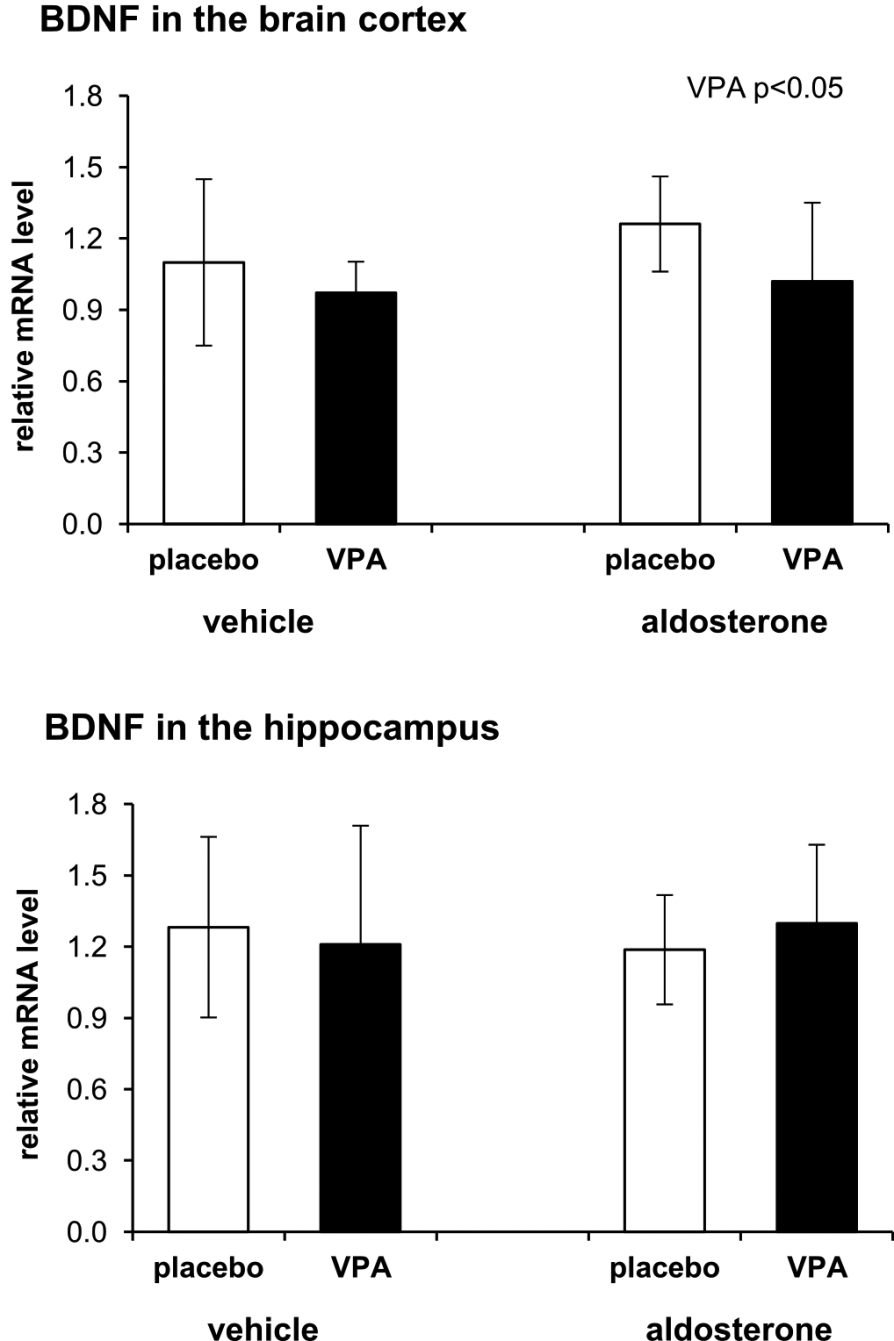
Effects of valproic acid (VPA) and aldosterone treatments on the brain-derived neurotrophic factor (BDNF) expression in the brain cortex and hippocampus. Each value represents the mean ± standard deviation (n = 10 rats/group). Statistical significance as tested by two-way ANOVA with the main factors of VPA treatment and aldosterone treatment. Presented results represent a relative value, namely a ratio between the quantity of measured gene and geometric mean of the quantities of reference genes (21).

Valproic acid treatment had a significant main effect on BDNF expression in the brain cortex (F_(1,34)_ = 4.39, *P* = 0.044). BDNF mRNA levels in the brain cortex were significantly decreased in animals treated with valproic acid compared with those treated with placebo. No significant main effect of aldosterone treatment or interaction between factors was observed (Figure 1). In the hippocampus, neither valproic acid nor aldosterone significantly affected BDNF expression (data not shown).

**Figure 2:**
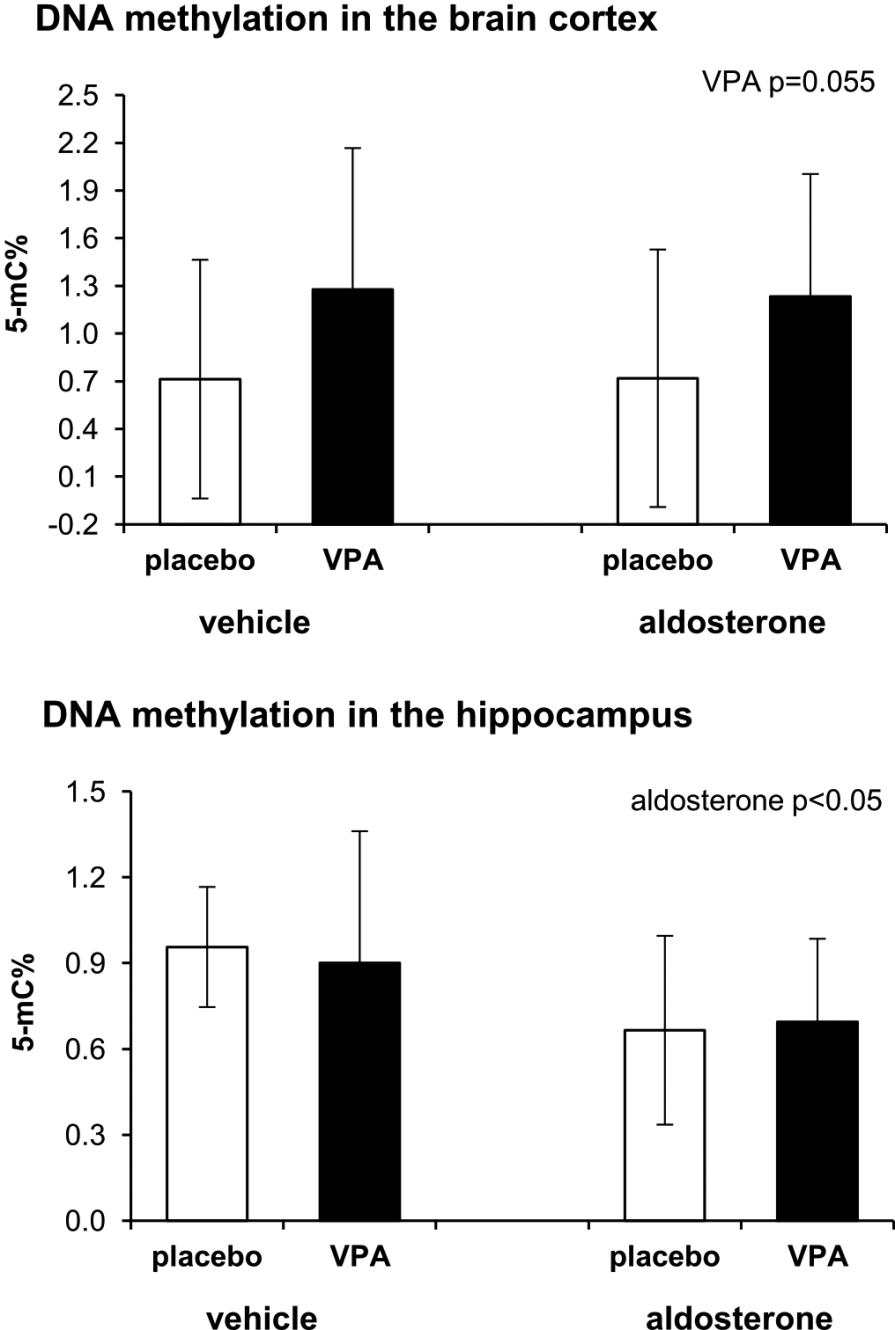
Effects of valproic acid (VPA) and aldosterone treatments on the global DNA methylation in the brain cortex and hippocampus. Each value represents the mean ± standard deviation (n = 10 rats/group). Statistical significance as tested by two-way ANOVA with main factors of valproic treatment and aldosterone treatment. 5-mC % – percentage of methylated DNA (5-methylcytosine).

There was a tendency toward an increase in global DNA methylation in the brain cortex of animals treated with valproic acid compared with those treated with placebo. However, the difference did not reach significance (two-way ANOVA F_(1,32)_ = 3.97, *P* = 0.055). No significant main effect of aldosterone treatment or interaction between factors was found (Figure 2). In the hippocampus, aldosterone treatment significantly influenced global DNA methylation (F_(1,34)_ = 4.42, *P* = 0.004), but valproic acid treatment did not. DNA methylation in the hippocampus was significantly reduced in animals treated with aldosterone compared with those treated with vehicle. No interaction between factors was observed.

**Figure 3:**
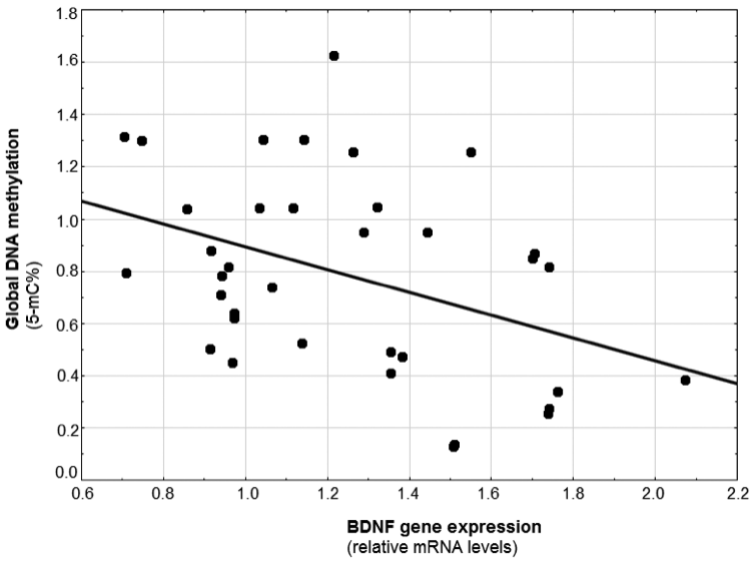
The negative correlation between the expression of brain-derived neurotrophic factor (BDNF) and global methylation in the hippocampus in the whole sample (r = - 0.41, *P* = 0.014). 5-mC % – percentage of methylated DNA (5-methylcytosine).

Pearson correlation analysis showed a significant negative correlation between BDNF expression and global methylation in the hippocampus in the group of vehicle-valproic acid treated animals (r = - 0.73, *P* = 0.042), but not in other groups (vehicle-placebo group: r = 0.37, *P* = 0.412; aldosterone-placebo group: r = - 0.43, *P* = 0.216; aldosterone-valproic acid group: r = - 0.61, *P* = 0.149). The negative correlation between the expression of BDNF and global methylation in the hippocampus was significant also in the whole sample (Figure 3). In the brain cortex, no significant correlations between the BDNF expression and global methylation were found (vehicle-placebo group: r = -0.55, *P* = 0.181; vehicle-valproic acid: r = -0.12, *P* = 0.785; aldosterone-placebo group: r = - 0.18, *P* = 0.614; aldosterone-valproic acid group: r = - 0.11, *P* = 0.786).

## DISCUSSION

In contrast to our hypothesis, two-week treatment with valproic acid had no impact on BDNF gene expression in the hippocampus but reduced BDNF mRNA levels in the brain cortex. Valproic acid treatment also marginally enhanced global DNA methylation in the brain cortex. An unexpected finding was a significant decrease in global DNA methylation in the hippocampus induced by aldosterone treatment.

Two-week treatment with valproic acid in the present model failed to modify BDNF mRNA levels in the hippocampus. Similarly to our findings, three-day valproic acid treatment also had no effect on BDNF mRNA levels in the hippocampus (22). However, the majority of other studies observed that valproic acid enhanced hippocampal BDNF expression (23-25). In these studies, treatment with valproic acid reversed an impairment of BDNF expression induced in animal models of bipolar disorder, Alzheimer’s disease, or facial nerve transection. Though single, acute stimulation of mineralocorticoid receptors affected BDNF expression in several brain regions (26), no changes in BDNF expression were induced in the present animal model of hyperaldosteronism. Other authors who also treated rats with valproic acid without previous impairment of cognitive functions or BDNF expression even observed a decrease in hippocampal BDNF protein levels (9,11). Thus, the action of valproic acid may depend on the extent of pathological changes at the time of treatment onset. Interestingly, a new anticonvulsant drug lacosamide, which has some mechanisms of action in common with valproic acid, induced dose-dependent reduction of the hippocampal expression of BDNF and its receptor (27).

In the present study, valproic acid treatment even decreased the gene expression of BDNF in the frontal cortex. The results of the few studies describing BDNF expression in this brain region are equivocal, reporting no changes (28) or reversal of previous impairment (23,25). A decrease in BDNF expression, as observed in the present study, was previously described only when valproic acid was injected directly into the specific region of the frontal cortex associated with enhancement of stress-related memory formation (29).

In the present study, valproic acid treatment did not change global DNA methylation in the hippocampus, and the slight increase in the frontal cortex failed to be significant. Studies on cell cultures demonstrated that valproic acid triggered active DNA demethylation (30,31). However, the information on the influence of valproic acid on DNA methylation in studies dealing with brain psychopathology is scarce (12). We found a significant negative correlation between BDNF mRNA levels and the percentage of methylated DNA in the hippocampus of valproate-treated control rats. This finding is in agreement with the results of experiments suggesting that pharmacological inhibition of DNA methylation is associated with increased hippocampal BDNF expression (32).

An original finding of the present study is a decrease in the global DNA methylation in the hippocampus induced by the aldosterone treatment. To our knowledge, there are no data on the effects of aldosterone on DNA methylation in the brain. Available are only studies on adrenocortical adenoma producing aldosterone in humans, in which the majority of genes were demethylated (33). Interestingly, aldosterone had a significant impact on global DNA methylation in the hippocampus and almost none in the brain cortex, which is consistent with high mineralocorticoid receptor densities in the neurons of the hippocampal formation (34). In contrast, valproic acid had a larger impact on global DNA methylation and BDNF mRNA expression in the brain cortex than in the hippocampus.

A limitation of the present study is that the experiments were performed in one sex only. Another limitation is possible variability in valproic acid dosing as the treatment was performed via drinking water.

Based on the results obtained and literature data, the effect of valproic acid on BDNF expression in the studied brain structures may depend on the extent of pathological changes present at the time of treatment onset. The negative correlation between BDNF expression and DNA methylation in the hippocampus of valproic acid-treated rats encourages further studies.
